# Rhythmic Physical Activity Intervention: Exploring Feasibility and Effectiveness in Improving Motor and Executive Function Skills in Children

**DOI:** 10.3389/fpsyg.2020.556249

**Published:** 2020-09-18

**Authors:** Spyridoula Vazou, Brenna Klesel, Kimberley D. Lakes, Ann Smiley

**Affiliations:** ^1^Department of Kinesiology, Iowa State University, Ames, IA, United States; ^2^Department of Psychology, Iowa State University, Ames, IA, United States; ^3^Department of Psychiatry & Neuroscience, University of California, Riverside, Riverside, CA, United States

**Keywords:** chronic exercise, executive processing, physical education, cognition, social, enjoyment, youth

## Abstract

**Introduction:**

Increasing literature has emerged investigating the importance of considering the qualitative characteristics of physical activity (PA) interventions and sports as well as considering the role of motor competence in the exercise–cognition interplay. The purpose of this pilot study was to examine the feasibility and effectiveness of a rhythmic PA intervention compared to a standard physical education program, on motor and hot and cool executive function (EF) skills.

**Methods:**

Children ages 6–11 were enrolled in one of the two programs: a rhythmic program (*n* = 22) and a physical education program (*n* = 17), both meeting for 30 min, twice per week, for 7 weeks. The rhythmic program emphasized moving to the beat of music and moving in various rhythmic patterns with whole body movements, clapping, and drumsticks. The children also created their own rhythmic patterns and socially engaged with other children by working in pairs and sharing their routines with the group. The physical education group engaged in ball skills, locomotor patterns, team sports, and moving through stations in small groups, with no emphasis on rhythm. Pretest and posttest measurements included measurement of balance (Movement ABC-2), cool and hot EF (Flanker, SWAN), and social factors, whereas throughout the implementation period data on affective valence, enjoyment, cognitive engagement, perceived exertion, and PA levels were collected at every lesson in both groups.

**Results:**

The rhythmic program used in this study was feasible, scalable, affordable, and able to be implemented with minimal preparatory time. Children in both groups (rhythmic and physical education) engaged in a similar level of PA and had similar positive experiences from the programs. Both groups improved in balance and cool EF, and there were significant correlations in the change scores between balance and cool EF, as well as between cool EF with hot EF and socio-emotional factors.

**Discussion:**

This study contributes to the literature by exploring the potential value of rhythmic programs as a vehicle in helping children develop motor and EF skills while deriving joy and positive social interactions from the program.

## Introduction

Promotion of physical activity (PA) in youth is acknowledged as a priority by many prominent public health organizations in the United States (Centers for Disease Control, and Prevention [CDC], 2010; Institute of Medicine [IOM], 2013). Organizations have emphasized the need to include more PA throughout the school day, with physical education as a central component in the implementation of Comprehensive School PA Programs (Centers for Disease Control, and Prevention [CDC], 2010; Institute of Medicine [IOM], 2013; Shape America, 2014). The ASCD (former Association for Supervision and Curriculum Development), a leading educational agency, in collaboration with the CDC adopted a joint goal to promote learning and health through the whole-school, whole-community, whole-child model (ASCD and Centers for Disease Control and Prevention [CDC], 2014). The ASCD’s Whole Child Initiative views the collaboration between learning and health as fundamental, emphasizing the need to shift the focus from narrowly defined academic achievement to one that promotes long-term development and success of children (ASCD and Centers for Disease Control and Prevention [CDC], 2014).

Compelling evidence demonstrates that PA is associated with improvements in both cognitive function and academic performance (e.g., Mura et al., 2015; Donnelly et al., 2016), which is supported by research describing the interconnection between motor and cognitive functions in children (e.g., Diamond, 2000; Van der Fels et al., 2015). Evidence indicates, however, that not all forms of PA benefit cognition equally (Diamond and Lee, 2011; Pesce, 2012; Diamond, 2015; Pesce and Ben-Soussan, 2016; Vazou et al., 2019b), and it has been suggested that “the degree to which the exercise requires complex, controlled, and adaptive cognition and movement may determine its impact on executive functions (EF)” (Best, 2010, p. 336). Researchers have argued that children who perform activities that are not challenging because of lack of progression in task difficulty do not challenge EF (Diamond and Lee, 2011) and that when instructional methods challenge learner’s thoughts and actions, cognition is enriched and maintained (Tomporowski and Pesce, 2019). Thus, there are likely a number of contextual factors, including instructional techniques, intervention content and structure that impact the degree to which a PA intervention improves EF.

A recent review and meta-analysis on different characteristics of PA (i.e., aerobic, motor skills, cognitively engaging activities, and all combinations of those facets) supported the significant positive effect of PA interventions on EF (0.46 effect size) and identified differences between the categorically different long-term PA interventions (Vazou et al., 2019b). However, as several researchers have suggested, the quantitative and qualitative characteristics of PAs, as well as unique elements within a PA program (e.g., type of movement, mental resources, skill acquisition, emotional activation) and contextual factors (including the physical and the social environment) may impact the effectiveness of those interventions (Tomporowski and Pesce, 2019; Vazou et al., 2019b). As Diamond (2015) recommended, the most effective interventions are likely those that, “(a) train and challenge diverse motor and executive function skills, (b) bring joy, pride, and self-confidence, and (c) provide a sense of social belonging (e.g., group membership) (p. 963).” Moreover, interventions may differentially affect two types of EF. “Cool EF” are those exhibited in “decontextualized and affectively neutral conditions” (Pesce et al., 2020), such as working memory, inhibition, and strategy use, while “hot EF” involve cognitive and emotional control in “contexts that generate heightened emotion” (Pesce et al., 2020), such as playing a game and making decisions in an emotionally charged environment. This research points to the importance of studying contextual factors associated with diverse PA interventions.

From a practical standpoint, PA interventions that are designed for youth also should consider the natural desire in children to explore new movements and skills. PA programs should challenge children using a wide variety of activities that focus on motor skill development and variability of practice (Myer et al., 2016; Pesce et al., 2019), while simultaneously providing the novelty and variety necessary to sustain interest and enjoyment (Sylvester et al., 2018). Positive affect and enjoyment predict PA behavior (Cox et al., 2008; Nasuti and Rhodes, 2013) and have been proposed to be associated with higher levels of engagement and self-regulatory skills (Isen and Reeve, 2005; Diamond, 2015; Brand and Ekkekakis, 2018). Recognizing the importance of engagement, National Standards in the United States (SHAPE America) include a physical education goal of providing enjoyable and varied PA that also enhances children’s motor skill development.

Tomporowski and Pesce (2019) highlighted common processes involved in PA and the performing arts that may benefit cognition. PA programs with a rhythmic or music component, such as dance or drumming, are hypothesized to improve EF (e.g., Diamond, 2014), as rhythmic movements challenge both cognitive and motor systems. They require mental effort to move with the tempo and require learning new rhythms and accompanying movements. However, little research has focused on the cognitive benefits of combining music and PA in an integrated intervention. Lakes et al. (2019) described expressive or rhythmic movement as having the potential to extend both the beneficial effects of music and PA, positing that music and movement interventions can provide multisystem learning opportunities characterized by embodied cognition. The embodied cognition perspective describes cognitive processes as embedded within the ways in which we interact within the world (Wilson, 2002), and cognitive improvements are likely strengthened when trained in such a context. Preliminary studies examining rhythmic PA programs and cognition include research by Lakes et al. (2019) examining the Creatively Able program (an intervention involving music, dance, and the engagement of children in creating choreography) in children with promising findings related to children’s engagement, enjoyment, and cognitive functions. Similarly, an intervention with music education was found to positively affect EF skills and academic achievement (through the mediating role of EF) in typically developing elementary children (Jaschke et al., 2018). More research on interventions with these features is warranted.

This pilot study was designed to evaluate the feasibility and effectiveness of a novel rhythmic PA program compared to a generalized physical education program, on motor and cognitive skills in children. We examined several qualitative and contextual features of the rhythmic PA program and their possible association with motor and cognitive skills. This feasibility study was designed to help researchers and practitioners determine whether a rhythmic intervention should be recommended for evaluation on a larger scale and potentially implemented as a PA program for youth during physical education or after-school. Our study goals were consistent with those recommended in intervention literature that emphasize the importance of early examinations of the feasibility of new interventions with a focus on acceptability, demand, implementation, practicality, and limited-efficacy testing (Bowen et al., 2009).

## Materials and Methods

### Recruitment and Participants

**FIGURE 1:**
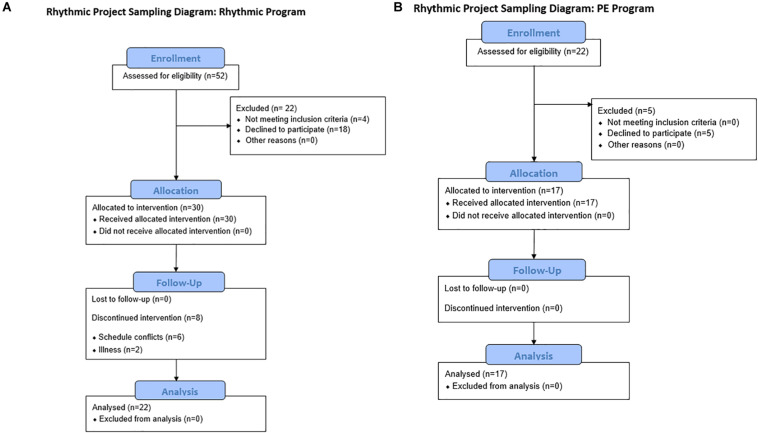
Study flowchart showing retention of participants in the rhythmic **(A)** and the PE **(B)** group.

A total of 39 children, ages 6–11 [*M* age = 7.69 ± 1.52 years; 21 males (53.85%)], participated in this study, with 22 in the rhythmic group and 17 in the physical education group. Table 1 includes the demographic characteristics of the participants in each group. The physical education group was recruited through a physical education program for homeschooled children offered through a university course by the lead researcher. The Rhythmic group was recruited from community advertisements and the university email list for faculty and staff. In addition to age, inclusion criteria were the parent’s ability to consent in English and the child’s ability to assent in English. Exclusion criteria included a DSM-5 diagnosis other than Developmental Dyslexia or Attention Deficit Hyperactivity Disorder (ADHD), an IQ below 80, or parental report of a history of developmental disability, intellectual disability, or brain trauma. Figure 1 shows the CONSORT flow diagram of enrollment for both groups. A total of 47 children were initially recruited, 17 in the physical education group and 30 in the rhythmic group. Six participants dropped out before the implementation due to scheduling conflicts, and two participants dropped out due to illness; all eight were enrolled in the rhythmic group. The study was approved by the university Institutional Review Board. Written informed consent and verbal assent were collected from parents and children, respectively.

**TABLE 1:** Demographic characteristics of participants in each program.

	**Physical Education (*n* = 17)**	**Rhythmic (*n* = 22)**
Sex	10 Male	11 Male
	7 Female	11 Female
Race/Ethnicity	15 Caucasian	15 Caucasian
	2 Asian	5 Asian
		1 African American
		1 Mixed
Average Age (years)	7.76 ± 1.64	7.64 ± 1.47
Average Height (cm)	134.33 ± 10.40	135.18 ± 10.95
Average Weight (kg)	31.34 ± 8.53	30.61 ± 8.28
Average BMI (kg/m^2^)	17.12 ± 3.17	16.44 ± 2.58
IQ Standard Score	107.76 ± 16.98	111.73 ± 15.15
IQ Percentile	62.34 ± 30.62	71.49 ± 27.71
Fitness Score	16.31 ± 10.37	14.29 ± 9.37

### Measures

#### Baseline Measures

For demographics, weight and height were measured privately with a body scale and a stadiometer. Body mass index was computed by dividing weight (in kilograms) by the square of height (in meters).

The Kaufman Brief Intelligence Test—Second Edition (KBIT-2; Kaufman and Kaufman, 2004). The KBIT-2 Non-Verbal Section is a measure of non-verbal intelligence (non-verbal IQ). Children select the picture on a flip card that best fits the matrix pattern pictured on the card. The matrices become progressively difficult, and when three consecutive mistakes occur, the test is stopped. Standardized scores are calculated based on United States norms for a given age group.

The Cardiovascular Endurance Fitness—PACER FitnessGram (4th edition; Plowman and Meredith, 2013) was administered in the gymnasium with a group of children. A black line and cones at the two ends of the gymnasium were used to mark the 20-m distance used for the task. The children were asked to line up at the start and to begin running from one side to the other following the prerecorded auditory signal, which becomes progressively faster. Children were asked to keep up with the cadence and stop when they can no longer keep up with it. The numbers of cycles each child completed were recorded by research assistants observing the test.

#### Pretest and Posttest Measures

The Movement Assessment Battery for Children-2 (M-ABC-2; Henderson and Sugden, 2007) balance subtest was administered individually. In this subtest, there are three tasks: (1) balancing on one foot on a balance board for up to 30 s (one trial on each leg), (2) walking on a line using tandem foot placement without stepping off the line for a maximum of 15 steps (2 trials), and (3) hopping in a controlled manner up to 5 continuous hops on a series of preplaced squares (2 trials). The scores are standardized so that a single subtest score is calculated and analyzed.

A modified version of the Rhythm/Beat Competence Assessment (Weikart, 2006) was administered individually. In this assessment, children were instructed to tap a pair of drumsticks together to the beat of the song. Children then listened to a standard song and tapped to the beat until the song ended, for a total of 64 beats. Observers measured rhythm/beat competence as the total number of times the tap matched the beat, with a maximum score of 64.

The Strengths and Weaknesses of ADHD-symptoms and Normal behavior (SWAN) rating scale (Swanson et al., 2012) was completed by parents to measure two factors: Attention (9 items; e.g., “sustain attention on tasks or play activities”) and Behavioral Control [9 items; e.g., “settle down and rest (control constant activity)].” The scale is appropriate for use from preschool age and beyond (Lakes et al., 2012). Each item is rated across a dimension using a seven-point response scale, ranging from “far below average” (rated in this study as 1) to “far above average” (rated in this study as 7). Cronbach’s alpha coefficients of internal consistency in the present study were acceptable, for both Attention (α = 0.84, α = 0.85) and Behavioral Control (α = 0.91, α = 0.89, for pre and posttest, respectively).

For EF, the computer version of Flanker Fish test (using Presentation software; Neurobehavioral Systems, 2012) incorporates the Standard Flanker (inhibition), Reverse Flanker (inhibition and working memory), and Mixed Flanker (inhibition, working memory, and cognitive flexibility) conditions, specifically modified for children (Diamond et al., 2007). In this computer version of the Flanker test, there are five fish shown and the child must “feed” the hungry fish by pressing the left or right key on the touch-screen, depending on which direction the target fish is/are facing. In the Standard Flanker task (17 trials), the fish are blue and the target fish is the middle one, requiring participants to inhibit attention to the fish positioned on either side. In the Reverse Flanker task (17 trials) that follows, the fish are pink, and the target fish are all but the middle one, facing in the same direction. This task demands inhibition of attention to the fish in the center plus flexible switching of mindset and attentional focus to the new rules. In the Mixed Flanker task (45 trials), administered last, either the blue or the pink fish may appear and the child must respond based on the correct rule. The stimulus presentation time was 2100 ms, and a practice block for each task preceded the actual test. For each task, both accuracy (percentage of correct answers) and reaction time (RT) were recorded.

Perceived competence toward exercise programs was assessed using the five items from the perceived competence subscale of the Intrinsic Motivation Inventory (McAuley et al., 1989), which has been previously used in elementary physical education and other youth program settings (Standage et al., 2005; Agans et al., 2019). An example item is “When it comes to playing physically active games, I think I am pretty good,” and the response scale ranged from 1 (“strongly disagree”) to 5 (“strongly agree”). The alpha coefficient of internal consistency in the present study was acceptable, for both pretest (α = 0.80) and posttest (α = 0.70).

Relatedness was assessed with the 6-item Relatedness to Others in Physical Activity Scale (Wilson and Bengoechea, 2010). Children responded to a number of items following a stem phrase (“In the exercise classes I participate in, I feel”) such as “I am included by others.” Each item was rated using a five-point response scale (1 = strongly disagree, 5 = strongly agree). Cronbach’s alpha coefficients of internal consistency in the present study were acceptable, for both pretest (α = 0.71) and posttest (α = 0.90).

#### Measures Across Intervention

##### Physical activity level

Accelerometer data and ratings of perceived exertion (RPE) were used as means of quantifying the physical activity intensity during all rhythmic and physical education lessons. A triaxial accelerometer (ActiGraph GT3X+; ActiGraph, Pensacola, FL, United States) was worn on the right hip over the clothes with an adjustable belt to objectively measure the total time spent in moderate or vigorous PA. ActiGraph monitors were downloaded using ActiLife v 6.11.4 software (ActiGraph, Pensacola, FL, United States) and were converted to 1-s epoch csv output files for further analysis. The percentage of time spent in moderate or vigorous PA during each 30-min lesson was estimated per child. Perceived exertion was assessed upon completion of each lesson using the boy and girl versions of the stepping Children’s OMNI RPE scale (Robertson et al., 2005), which ranges from 0 (not tired at all) to 10 (very, very tired).

##### Fidelity checklist

A fidelity checklist was developed to assess whether the PA programs were implemented as originally planned (Table 2). Specifically, the checklist assessed whether the rhythmic program provided opportunities for a variety of rhythmical gross motor actions with music, sounds, and manipulatives as well as social interactions. Similarly, the focus of the physical education lessons was assessed, which targeted working on all fundamental motor skills and fitness components, without specific emphasis on rhythmic movement.

**TABLE 2:** Fidelity checklist for physical activity programs.

**Rhythmic**					**Physical Education**				
**Focus**	**Music**	**Sounds/Singing**	**Mimic/Clapping/Sticks**	**Partner work**	**Body Movement (*non- locomotor, locomotor*)**		**Manipulative (*throw/catch, dribble, kick, volley*)**	**Locomotor and Tumbling**	**Fitness (*obstacle courses*)**
Percent	100%	71%	100%	78%	100%		57%	21.5%	21.5%

*For the Rhythmic lessons, all 4 components were targeted per lesson. For the PE lessons, the focus was on one main category per lesson.*

##### Process evaluation measures

The pictorial Feeling Scale (FS; Hardy and Rejeski, 1989), as adapted for children by Hulley et al. (2008), was used to measure affective valence from the experience in the PA programs, completed at the end of each lesson. The Feeling Scale is an 11-point single-item bipolar scale, asking children “How do you feel right now?”, with scores ranging from -5 (“Very bad”) to +5 (“Very good”) accompanied by gender-specific drawings ranging from a sad face (-5) to a happy face (+5).

Enjoyment from each lesson was measured by asking children how much they enjoyed the lesson with a 5-point single-item asking “How fun was the lesson today?” Children rated their enjoyment on a Likert scale, ranging from 1 (not at all fun) to 5 = very fun. This item was added in addition to the pictorial Feeling Scale to make it easier for the younger children to respond on how they felt about the lessons.

Cognitive engagement during each lesson was rated using a 5-point single-item asking, “How much did you use your brain to think during the lesson?” Children rated their cognitive engagement on a scale ranging from 1 (not at all) to 5 (very much) immediately upon completion of the lesson. The item was developed for the purposes of this study based on previous work on cognitively engaging PAs in youth (Schmidt et al., 2016).

Engagement during the lesson was observed by trained research assistants. The level of engagement was scored as the degree the children listened to and followed the program instructions throughout the 30-min lesson, with 1 indicating little engagement, 2 indicating moderate engagement, and 3 indicating strong engagement.

The motivational climate created by peers for the rhythmic program was measured with a modified version of the Peer Motivational Climate in Youth Sport Questionnaire (Ntoumanis and Vazou, 2005). The peer climate included 8 items from the task-involving motivational climate higher-order factor. Children responded to items assessing the degree to which their peers “helped each other improve,” “said nice things when I tried,” etc. In order to measure how supported children felt by the teacher, three additional items on teacher support (from the Learning Climate Questionnaire, Williams and Deci, 1996) were included in our research: “my teacher provided me with choices,” “my teacher encouraged me to make my own moves in the program,” and “my teacher understood me.” Cronbach’s alpha coefficients of internal consistency in the present study were acceptable, for both peer climate (α = 0.89) and teacher support (α = 0.70). The motivational climate and teacher support were administered at the end of the implementation period for the rhythmic group.

In addition to quantitative process evaluation data, five open-ended questions were developed to measure parent and child perceptions of the rhythmic program. The questions referred to the overall experience of children, what children liked or did not like as much, what the parents thought about the program, and any suggestions for changes if the program was offered in the future. The questionnaire was completed anonymously and placed in a sealed box available at the waiting area of the gymnasium.

### Procedure

Before the 7-week implementation period, all children engaged in two pre-session visits at the researchers’ university laboratory and gymnasium, lasting approximately 1 h each, for the collection of the consent forms, baseline, and pretesting assessments. Upon completion of the programs, a 1-h posttest evaluation was conducted. All testing was done individually, except for the PACER.

The intervention sessions for both the rhythmic and physical education groups took place in the gymnasium at the researcher’s university and were scheduled at approximately the same time of day (between 2:00pm and 5:30pm). All children attended two 30-min sessions per week, for seven weeks. All lessons were delivered by the first two researchers with the assistance of trained undergraduate students. Upon entry into the gymnasium, each child was outfitted with an accelerometer and immediately upon completion of the lesson, participants completed the single-item measures (affective valence, exertion, cognitive engagement, enjoyment) individually, and the accelerometers were removed. During each session, trained research assistants recorded observations for the fidelity assessment and of the students’ engagement.

#### Rhythmic Program

For the rhythmic program, three separate time slots were offered to accommodate different schedules, with about eight participants in each session. Active learning of rhythmic gross motor actions in response to different songs was the primary activity. The rhythmic session began with rhythmic education in which participants learned to define a beat and identify the types of musical notes the beats hit (e.g., What is a beat? Eighth note, quarter note, half note, whole note?). Participants then learned to clap, jump, hop, walk, run, bounce a ball, and drum to the beat in different musical notes. After learning and practicing the basics, participants learned movement sequences set to steady, easy-to-follow, 4-count beats, with and without music. Each sequence emphasized moving precisely to the beat and incorporated full body movements and drumming. Participants were asked to count the beat of the music loudly while moving for better comprehension. The creative component of the lesson plans included moving freely to the beat of the music and creating new sequences to teach the other participants. The social component of the lesson plans included performing the movement sequences while facing each other, switching positions with each other during the sequence, creating new sequences with others, and learning new sequences from the other participants. A more advanced social rhythmic activity included tinikling, a Philippine inspired activity in which two participants adorned elastic strips to their ankles and jumped in tandem together twice and apart twice while other participants jumped in and out of the center of their strips without touching the strips.

Each session included progression in difficulty, with (a) warm-up activities involving moving across-the-room with different rhythmical locomotor skills and bouncing stability balls without music first and then with music, (b) movement patterns taught by the instructor, and (c) creative movement patterns developed by the participants alone or with a partner. Each rhythmic lesson plan tasked high cognitive load on working memory, attention, and executive functions. The rhythmic intervention utilized to a large extent the Drums Alive^®^ program^1^ that provides training as well as ready-to-use resources (activities, videos, and music) that are low cost and easy to use with relatively limited preparation time (preparation time always depends on the experience of the instructor; the more experience one gets, the less preparation time is needed). The main equipment utilized in the rhythmic program were stability balls, buckets as a base, and drumsticks (for the drumming activities), as well as poly-spots and stretch bands (for the jumping activities).

#### Physical Education

Two physical education sections were offered at the same time, with children grouped dependent on age. The PE classes were taught as a larger teaching opportunity for undergraduate students offered to homeschooled children in the community, with only a portion of enrolled students participating in the study. The physical education lessons are characterized as an active learning environment for gross motor actions, including developmentally appropriate activities on locomotor, non-locomotor, manipulative skills, and fitness. None of the physical education activities emphasized moving to the rhythm of music. The social component of the lesson plans included traveling to stations in small groups, playing team sports, and working together to complete a sequence of movements. Each physical education lesson tasked some cognitive load on working memory, attention, and EF.

### Data Analysis

Data were analyzed using the Statistical Package for Social Sciences (IBM SPSS Statistics 26). The primary purpose of the study was to compare changes in motor and EF skills as a result of two treatments, namely, rhythmic exercise and physical education. Mixed-plot MANOVAs were conducted with two groups (intervention, comparison) for the between-subject factor and two time points (pre, post) for the within-subject factor, separately for each set of outcome variables. ANOVAs with a between-subject independent variable (group: rhythmic, physical education) were conducted for the process evaluation variables. Secondly, in order to examine associations between motor, hot and cool EF skills, and social–emotional characteristics of the program, three separate sets of correlations were conducted: (1) motor with hot EF and social–emotional factors; (2) motor with cool EF; and (3) hot EF and social–emotional factors with cool EF. For the correlations, the change scores for balance, Flanker tasks, and SWAN factors were calculated.

Scores on the Flanker tasks were filtered, and values were discarded according to the following criteria: (a) if they represented practice trials, (b) the first testing trial, (c) if RT < 250 ms, and (d) if they were outside 2 SD from the mean reaction time per task/per participant. Reaction time was quantified only for correct trials. Response accuracy was quantified as the proportion of correct responses relative to the number of trials administered. Repeated MANOVAs (group as the between-subject variable and time as the within) were conducted for the Standard, Reverse, and Mixed Flanker, with two dependent variables, namely, reaction time (in ms) and accuracy (%). Statistical significance was accepted at a level of *p* < 0.05. Effect sizes for differences between means were calculated using Cohen’s *d*.

## Results

### Intervention Fidelity and Feasibility of PA Programs (Rhythmic and PE)

#### Fidelity of Programs

The qualities of the rhythmic and the physical education programs are presented in Table 2. As shown in the Table, each of the rhythmic lessons focused on mimicking sounds with clapping or drumming sticks, listening to different beats of the music, and moving to the beat with locomotor (e.g., walking, jumping) or non-locomotor skills (e.g., bending, twisting). The majority of the lessons included working with a partner or a small group (78%) and singing or repeating sounds (71%; e.g., counting aloud) to the beat. For the physical education program, the focus of the majority (57%) of the lessons was on manipulative skills (e.g., passing, catching, dribbling, kicking, volleying), without excluding fitness lessons (obstacle courses and fitness stations), tumbling (e.g., rolling), and locomotor (e.g., dodging, galloping, jumping) skills.

#### Feasibility of Programs

The process evaluation data provide context about factors that may potentially influence implementation and outcomes. The descriptive statistics for the characteristics of the Rhythmic and PE programs are presented in Table 3. As evidenced, children in both groups had high attendance (>86%), with absences per child ranging from zero to four (Mean = 1.4, *SD* = 1.21). *T*-test comparisons showed no significant differences between groups for each of the process evaluation variables, except observed engagement. Specifically, data collected from each lesson showed that in both groups the children felt fairly good to good at the end of the lessons, perceived lessons as both fun and tiring, and believed they were cognitively engaged during the lessons. Further, there were no significant group differences in the MVPA levels during lessons or between students’ perceptions of their competence and relatedness at the end of the implementation period. The only significant difference between groups was found on observed engagement, with the children in the PE group showing greater engagement during the lesson.

**TABLE 3:** Descriptive statistics and baseline differences for the qualitative characteristics of physical activity programs.

**Characteristics**	**Rhythmic**		**Physical Education**		***t*-test (*p*-value)**	**Rating Scale/**
	***M***	***SD***	***M***	***SD***		**Measure**
Attendance^≠^	86.36%	9.46%	92.86%	5.74%	–	Yes, No
MVPA^¥^	40.68	7.69	42.01	5.41	0.37 (0.548)	%
Perceived Exertion^¥^	4.00	2.20	3.08	2.20	1.68 (0.203)	0-10
Affective Valence^¥^	2.45	1.97	2.35	1.06	0.03 (0.859)	(−5) – (+ 5)
Enjoyment^¥^	3.65	0.95	3.81	0.63	0.34 (0.336)	1–5
Cognitive Engagement^¥^	3.78	0.10	3.48	0.77	1.11 (0.299)	1–5
Observed Engagement^¥^	2.51	0.44	2.98	0.06	**18.14 (0.000)**	1–3
Relatedness*	3.81	0.86	3.99	0.74	0.42 (520)	1–5
Perceived Competence*	4.02	0.62	4.21	0.46	1.003 (0.324)	1–5
Peer Climate*	3.74	0.76	–			1–5
Teacher Climate*	4.26	0.61	–			1–5
Rhythmic Performance^€^	6.24	17.06	0.00	3.24	1.16 (0.293)	1–64

^≠^At the group level; ^¥^Average scores from 14 lessons (7 weeks, twice per week); *Measured post implementation; ^€^Measured at pre–post (change is provided). Values in bold show the significant results.

Rhythmic performance was measured before and after the implementation period to measure rhythmic skills, which were only taught in the rhythmic intervention program. Even though there was no significant difference between the two groups, the effect size for the rhythmic program was small-to-moderate (*d* = 0.42) whereas for the physical education program was null (*d* = 0.00). However, the large SD for the rhythmic group shows variability among children, suggesting that there may be substantial individual differences in this outcome or possible measurement error with the specific scale.

#### Qualitative Program Evaluation

Fourteen parents provided anonymous qualitative feedback reporting their impressions of the rhythmic program. All parents reported that their children liked the program and the activities, indicating that many children practiced them at home alone or with siblings. Some parents commented that their child’s favorite parts of the intervention were working with a partner and having opportunities to be creative. Some students favored the drumming activities while others expressed that drumming was their least favorite activity. All parents reported that they really liked the program, and several parents expressed that they would not recommend any changes for the program. The suggestions for changes if the program was offered in the future were that it be provided more frequently or for a longer period of time, include more choices for students, and add instant activities for when children entered the gym to minimize waiting time (As this was a research study, instant activities were not offered in order to keep the duration of the lessons consistent for all participants).

### Intervention Effects on Motor and Executive Function Skills

Independent sample *t*-tests revealed no baseline group differences across all outcome variables. Mean scores, standard deviations, and pretest–posttest effect sizes for all outcome variables are presented in Table 4.

**TABLE 4:** Descriptive statistics (M, SD, ES) for motor, hot and cool executive functions.

**Rhythmic Program**				**Physical Education Program**		
**Task**	**Pretest *M* (*SD*)**	**Posttest *M* (*SD*)**	**ES**	**Pretest *M* (*SD*)**	**Posttest *M* (*SD*)**	**ES**
Balance	25.72 (7.59)	28.33 (7.48)	0.35	24.92 (7.74)	29.25 (7.15)	0.58
***Flanker—Accuracy***						
Standard	83.16 (9.48)	85.83 (5.93)	0.34	83.05 (14.39)	87.55 (3.53)	0.43
Reverse	78.34 (11.82)	86.10 (7.38)	0.79	74.74 (17.62)	84.43 (5.86)	0.74
Mixed	67.88 (15.57)	81.42 (12.30)	0.97	69.41 (16.58)	80.52 (8.33)	0.85
***Flanker—RT***						
Standard	1246.09 (207.87)	1093.22 (175.45)	0.79	1325.41 (217.96)	1122.88 (126.89)	1.14
Reverse	1311.18 (269.34)	1155.91 (249.29)	0.60	1361.23 (179.45)	1112.12 (169.97)	1.43
Mixed	1372.23 (168.36)	1288.54 (134.43)	0.55	1358.82 (130.52)	1300.41 (124.08)	0.46
***SWAN***						
Attention	4.58 (0.63)	4.72 (0.80)	0.19	4.58 (0.52)	4.51 (0.52)	−0.13
Beh. Control	4.37 (0.94)	4.41 (0.93)	0.04	4.98 (0.93)	4.67 (0.85)	−0.35

#### Balance (MABC-2)

The 2 (group) by 2 (time points) ANOVA showed a significant main effect of time [*F*(1,28) = 8.98, *p* = 0.006, η^2^ = 0.24] but no significant group or group by time interaction. Children in both groups improved their balance from before to after the 7-week period of implementation, with medium effect sizes for the PE group (*d* = 0.58) and small-to-medium effect sizes for the rhythmic group (*d* = 0.35).

#### Executive Functions (Flanker)

Repeated MANOVAs and the follow-up ANOVAs showed a significant main effect of time for the Standard [on RT: *F*(1,37) = 53.24, *p* < 0.001, η^2^ = 0.59], the Reverse [on both reaction time and accuracy: *F*(1,37) = 64.05, *p* < 0.001, η^2^ = 0.63; *F*(1,37) = 13.89, *p* = 0.001, η^2^ = 0.27, respectively], and the Mixed Flanker [on both reaction time and accuracy: *F*(1,37) = 15.14, *p* < 0.001, η^2^ = 0.29; *F*(1,37) = 31.14, *p* < 0.001, η^2^ = 0.46; respectively]. No main effect of group or interaction effects were evident. In all Flanker tasks, both groups improved significantly over time with large effect sizes (Table 4). Specifically, on accuracy, the effect sizes were larger as the difficulty of the Flanker tasks increased for both groups, with the largest effect sizes observed on the Mixed Flanker (*d*’s = 0.97 and 0.85, for rhythmic and physical education, respectively). For reaction time, the effect sizes were large (*d*’s = 0.79 and 1.14, for rhythmic and physical education, respectively) on the Inhibitory control (Standard Flanker) and medium on the other tasks (*d*’s = 0.46–0.60) with the exception of the Reverse Flanker, where the largest effect size observed in the physical education group (*d* = 1.43).

#### Attention and Behavioral Control (SWAN)

Results from the mixed-plot ANOVAs showed no significant main or interactive effects on Attention and Behavioral Control. However, for the Rhythmic group the effect size was small and positive on Attention (*d* = 0.19) but null on Behavioral Control (*d* = 0.04), whereas for the physical education group the effect size was small and negative for both Attention and Behavioral Control (*d* = −0.13, *d* = −0.35, respectively).

### Relationships Between Motor Skills, Hot and Cool EF, and Social–Emotional Factors

The results of the correlations between change scores for motor skills (e.g., balance), hot EF and social–emotional characteristics of the programs (SWAN ratings, affective valence, enjoyment, relatedness), and cool EF (Flanker accuracy scores and reaction time) are presented in Table 5. Between balance and cool EF, five of six correlations were in the hypothesized direction (positive), with two reaching statistical significance (e.g., balance with the Reverse Flanker accuracy and Mix Flanker reaction time). Balance was not correlated significantly with measures of hot EF (SWAN scores). Between change scores for measures of cool and hot EF, and social–emotional factors, significant positive correlations were found between the Reverse Flanker Accuracy score and children’s self-rated Affective Valence, Enjoyment, and Cognitive Engagement during intervention sessions. No significant correlations were evident for Flanker tasks with Attention and Behavioral Control (SWAN scores). However, due to the small sample size these results should be interpreted with caution.

**TABLE 5:** Correlations among motor, hot and cool executive functions, and characteristics of the physical activity programs.

	**Balance**	**Affect**	**Cognitive Engagement**	**Enjoyment**	**Exertion**	**Relatedness**	**Teacher Climate^≠^**	**Peer Climate^≠^**	**MVPA**
Balance	–	–0.187	−0.116	−0.219	−0.233	0.017	−0.032	0.288	
Flk Stand AC	0.145	0.366*	0.540**	0.460**	−0.276	0.201	0.221	0.179	0.105
Flk Rev AC	0.443*	−0.042	0.005	0.226	0.101	0.085	0.102	0.298	0.043
Flk Mix AC	0.297	−0.115	−0.053	−0.083	0.104	−0.010	0.102	−0.107	−0.209
Flk Stand RT	0.293	−0.160	0.055	0.045	0.048	0.027	0.072	−0.064	0.045
Flk Rev RT	−0.016	−0.124	−0.071	−0.059	0.161	−0.112	−0.238	−0.439	−0.061
Flk Mix RT	0.504**	0.054	0.037	−0.022	−0.099	−0.065	−0.120	−0.052	−0.109
Attention	−0.171	−0.014	0.139	0.027	−0.101	0.086	0.491*	−0.167	0.195
Beh. Control	−0.265	−0.082	0.016	−0.110	−0.087	0.024	0.332	0.084	−0.086

*N = 34. Change scores were calculated for Balance and Cognitive Function tasks. ^≠^*N* = 17. Values in bold show the significant results.*

#### Exploratory Analyses: Social Context as an Important Contextual Factor

To gather preliminary data on potential social contextual factors that might impact outcomes in a PA intervention, we examined correlations between teacher climate, peer climate, and EF outcomes among the children in the Rhythmic group (*n* = 17). Teacher climate was positively correlated with Flanker accuracy scores, Attention, and Behavioral Control, and the correlation with Attention (*r* = 0.491) reached statistical significance. None of the correlations between peer climate and EF change scores were statistically significant. Again, given the small sample size, results should be interpreted with caution.

## Discussion

The aim of this pilot study was to examine the feasibility of a rhythmic PA program as well as the extent to which a 7-week rhythmic PA program, compared to a generalized physical education program, impacts motor and EF skills in children. Children in the rhythmic group participated in a novel PA program focused on rhythmic movement patterns with music and manipulatives over fourteen sessions (two 30-min sessions per week). As research on rhythmic PA programs is newly emerging, our focus was primarily on experiences derived from the implementation of a rhythmic PA program with a secondary focus on potential effects on motor and cognitive outcomes in order to generate hypotheses for a full-scale randomized intervention study.

### Intervention Fidelity, Acceptability, and Feasibility

First, fidelity checklists demonstrated that teachers were able to deliver the content of both the rhythmic and physical education lessons as intended. Second, children’s experiences within and perceptions of the rhythmic program were measured using a variety of methods throughout each session, as well as at the end of the implementation period with a parental open-ended questionnaire. As the feasibility assessment showed, children enjoyed participating in the rhythmic intervention, perceived it to be cognitively engaging and a little tiring physically, and perceived both their peers and the teacher to be supportive and task-involving.

The inclusion of music and rhythmic movement patterns within an environment characterized by positive social interactions and task-involving teacher and peer climates made this PA intervention unique. Ours is one of the first studies to examine the social context of a PA intervention and supports the need for future consideration of instructional and peer climates. It is well-established that peer interactions affect children’s motivation and behavior in school (Wentzel and Ramani, 2016), as well as in youth PA contexts (Harwood et al., 2015). According to Self-Determination Theory (Deci and Ryan, 2000), relatedness is one of the three basic psychological needs, along with competence and autonomy, the satisfaction of which leads to intrinsic motivation and adaptive motivational outcomes, such as effort and commitment. Relatedness refers to a sense of feeling connected to others, supported, and valued by significant others (Deci and Ryan, 2000). Youth PA programs that support the need for relatedness, especially by peers, reflect higher levels of engagement, intrinsic motivation, enjoyment, and perceived competence (Ntoumanis et al., 2007; Hein and Jõesaar, 2015; Sparks et al., 2015). The role of peer relatedness on cognitive outcomes remains largely unexplored. Diamond and Ling (2015) predicted that successful interventions on EF skills are likely to be those that create feelings of belonging to a group with an important shared goal, whereas PA without cognitive challenge and lack of any social component appear not to improve EFs. Future PA intervention research should address peer and teacher climates to extend our understanding of how climate may impact both engagement in PA and outcomes.

The inclusion of music is a strategy commonly used to facilitate positive affective responses during exercise even when the intensity is vigorous (Deforche and De Bourdeaudhuij, 2015; Vazou et al., 2019a), and results from this study extend prior findings by illustrating children’s enjoyment of physical activities performed to music. The qualitative data from parents were very encouraging, suggesting that the children had positive experiences in the program, especially from the partner work and the creative components of the tasks, features of the intervention that should be promoted more extensively in future rhythmic programs. Suggestions from parents emphasizing the continuation of the program with a higher dose and frequency were heartening as they demonstrated demand for and interest in this type of intervention. Moreover, the use of a commercial program (Drums-Alive) with already developed resources (music, lessons), relatively low-cost equipment, and limited preparation time further illustrate that the rhythmic program is practical and could be easily delivered by PE teachers or other trained educators.

### Intervention Promotion of Skill Acquisition and Cognition

Importantly, our intervention was uniquely designed to focus on learning to listen and practice rhythmic movement, a skill that has not been examined systematically in the chronic PA intervention and cognition literature. Results showed that the 7-week rhythmic program improved children’s rhythmic skills (*d* = 0.42) and had a positive and meaningful effect on balance (*d* = 0.35), accuracy and response time on the Flanker tasks (*d*’s = 0.34–0.97), and parent-rated attention (*d* = 0.19). Similarly, Lakes et al. (2019) found that music and rhythmic movement, through the Creatively Able program, resulted in group-level reductions in Stereotyped and Compulsive behaviors for children with Autism Spectrum Disorder. The inclusion of music as an element of a PA intervention is further supported by research in typically developing elementary children showing that music education has a positive effect on EF skills and academic achievement (Jaschke et al., 2018). Thus, rhythmic programs, in addition to having a potential beneficial effect on motor and EF skills, also have the potential to benefit academic achievement and literacy skills, especially in early years of a child’s development (Bhide et al., 2013; Linardakis et al., 2013; Nelson, 2016). It has been suggested that rhythm and language show common developmental elements (Patel, 2003) and share some of the same auditory mechanisms (Degé and Schwarzer, 2011) as well as neural and cognitive resources that are necessary for both reading acquisition and music/rhythm understanding (Tierney and Kraus, 2013). Identifying factors that could potentially mutually help EF and literacy skills is particularly important considering the high percentage (5–17%) of schoolchildren who do not become fluent readers, even with explicit instruction (Shaywitz, 1998). This research on music and education supports the hypothesis that music could be an important contextual factor to include in PA interventions in order to synergistically improve executive functions and academic outcomes.

A process evaluation designed to control for potential covariation of PA intensity and other qualitative characteristics across the two programs (rhythmic and physical education) indicated that the programs did not differ in measures of PA intensity or children’s perceptions of the experiences. The only process variable that was significantly different between the rhythmic and the physical education groups was the level of observed engagement of the children during the lessons. While engagement was high in both groups (means across 14 lessons of 2.51 and 2.98 on a 3-point scale, for the rhythmic and physical education groups, respectively), it was significantly higher for the physical education group. This observation may be attributed to the lack of familiarity with the novel and possibly unusual rhythmic motor patterns that were practiced in the rhythmic program (e.g., drumming on stability balls, tinikling), whereas the physical education program included activities that the children were more familiar with, such as tag games, dribbling, kicking, and passing. The difference in students’ observed engagement may also be explained by the difficulty level of the tasks expected to be learned during the novel rhythmic lessons. It is possible that motor learning in the rhythmic program was more difficult because the tasks and motor patterns were new to the children. Even though the lessons were intentionally developed to provide variability of practice and to challenge the rhythmic and coordinative movements in order to enhance cognitive engagement and avoid automaticity (Pesce et al., 2013b, 2019), rhythmic programs may require either a longer duration of practice for successful performance and learning or increased scaffolding, hypotheses that need to be tested in future studies.

Our data showed that children in both programs improved significantly from pre- to post-intervention on balance and hot/cool EF skills, with no differences in changes between the two programs (i.e., one was not better than the other). Specifically, all children improved on balance, with the effect sizes being small-to-medium and medium for the rhythmic and the PE programs, respectively. Similar to our study, the enriched physical education program by Pesce et al. (2019) that was centered on being cognitively engaging and providing variability of practice showed improvements in all motor coordination skills in children, including balance.

Moreover, children in both groups improved on measures of cool EF, including reaction time in all three computerized tasks of EF (Standard, Reverse, Mixed Flanker) and on accuracy for both the Reverse and Mixed Flanker tasks. Notably, for accuracy, as the difficulty level of the Flanker tasks increased (by focusing on more than one executive process), the effect sizes were larger for both groups (*d*’s = 0.97 and 0.85 on the Mixed Flanker, for the rhythmic and physical education, respectively). Similarly, large effect sizes were found on reaction time for the Standard Flanker (inhibitory control) for both groups as well as on the Reverse Flanker (inhibition and cognitive flexibility) for the physical education group (1.43).

The improvement on tasks requiring all three cool executive processes (inhibition, cognitive flexibility, and working memory) suggest that developmentally appropriate PA programs of moderate-to-vigorous intensity (40–42%) that include cognitive engagement and variability of practice on fundamental motor skills can target a broad set of EF skills in children. Previous studies with rich PA interventions have also demonstrated positive effects on EF skills (Lakes and Hoyt, 2004; Chang et al., 2013; Lakes et al., 2013; Pesce et al., 2013a; Crova et al., 2014; Schmidt et al., 2015). However, in some studies, the results were not entirely consistent across all EF skills, suggesting differential associations between specific aspects of the PA programs and specific executive processes. For example, Schmidt et al. (2015) found that changes in inhibition were similar for all experimental groups (team games, aerobic, or traditional physical education) regardless of the intensity of the programs or the level of cognitive engagement, and working memory improved similarly for team games and the aerobic program but not in the physical education group. In contrast, cognitive flexibility improved significantly more for the team games program compared to the other two programs.

In our study, both accuracy and reaction time on all three cool EFs improved for both groups, but the present data are limited in that it cannot answer whether the improvements were simply due to maturation or to the characteristics of the PA programs. However, our results are in line with the conclusions of a recent meta-analysis (Vazou et al., 2019b), in which when a PA intervention, regardless of its qualitative characteristics (i.e., aerobic, motor skill, cognitively engaging), was compared to traditional physical education, which is known to be beneficial for children, the effects on the cognitive outcomes were of similar magnitude, with the pooled effect sizes being close to zero, and the largest effects being observed when comparing an intervention to no treatment. Thus, our design choices—using physical education as a control or comparison intervention—reduced our expectations for detecting large effects. These findings support the overall benefits of high-quality physical education delivered within the context of a research study.

### Associations Between Motor Skills, Hot and Cool EF, and Qualitative Characteristics of the Programs

In this study, the associations between balance (a measure of motor skill), cool EF and hot EF, and social–emotional factors were also examined. The results showed significant positive correlations between balance and measures of cool EF, specifically accuracy on the Reverse Flanker task (Inhibition and Cognitive Flexibility) and reaction time on the Mixed Flanker task (Inhibition, Working Memory, Cognitive Flexibility), but not with the Standard Flanker task that only involves inhibition. Gross motor skills have been found to significantly relate to specific EFs, such as visuospatial working memory and response inhibition (van der Fels et al., 2019). Balance was not correlated significantly with measures of hot EF and social–emotional factors.

These results are consistent with prior research examining relationships between cool and hot EF and motor skills, which have demonstrated positive relationships between EF and gross motor skills, but failed to find the same relationship when looking specifically at balance. In a school-wide sample of 207 children, Lakes (2013) found that observer ratings of gross motor control (e.g., coordination, athleticism) were significantly correlated with measures of cool EF (working memory and mental math tasks) as well as a measure of hot EF (teacher ratings of problem behavior); moreover, Lakes (2012) reported significant correlations between observer ratings of motor control and observer ratings of cognitive and emotional control when completing a challenge course. When the differentiating effects of specific motor skills on EF were examined, Pesce et al. (2019) found that ball skills, but not balance, had a mediating role on the effect of a cognitively engaging physical education intervention on inhibition. Additionally, in a cross-sectional study with young tennis athletes, it was demonstrated that a longer duration of game-based exercises was associated with better reaction time in inhibitory control, and coordination training was related to accuracy in working memory (Ishihara et al., 2017). In sum, there is evidence that gross motor skills are associated with cool and hot EF, and studies examining specific motor skills have demonstrated some differences in the relationships with selective EFs when specific motor skills are examined. In future research, it would be helpful to differentiate between specific motor skills and cool and hot EF to further our understanding of how these factors are related as well as how they might be impacted by PA interventions.

Positive significant correlations were also evident between cool EF (i.e., accuracy on Standard Flanker) and affective valence, cognitive engagement, and enjoyment of the PA programs. Deriving satisfaction from a PA program and experiencing positive affect has been proposed to be associated with higher levels of engagement and self-regulatory skills (Isen and Reeve, 2005; Diamond, 2015; Brand and Ekkekakis, 2018). It is noticeable that significant positive correlations were observed only between affective valence, cognitive engagement, and enjoyment (qualitative characteristics of the PA programs) with the Standard Flanker, which is the easiest task as it is only tapping attention and inhibitory control without requiring working memory and cognitive flexibility demands. It is possible that these qualitative characteristics may have a larger impact in changes of the “simpler” EFs and not in EF tasks involving greater complexity (including working memory). However, due to the preliminary nature of this study, this finding needs to be further explored in the future. Lastly, a supportive teacher motivational climate significantly correlated with attention, suggesting that instructors are likely another important contextual factor in a given intervention that deserves further consideration. Future PA intervention research should include measures of teacher climate in order to assess whether or not teacher climate may mediate intervention effects.

### Limitations and Future Recommendations

As this was a pilot study, several limitations should be taken into consideration both in planning future research and in interpreting the present results. These results clearly need to be replicated with a larger sample, and with a randomized sample. The lack of an inactive control group (wait list or no intervention) limits interpretation of the results. In addition, due to limited resources and time in the comparison group (most children had a limited time availability due to other after-school activities that restricted the number of measures we could use), balance was the only measure of motor skills that was evaluated. A wider assessment of motor skills, including both gross and fine motor skills (manual dexterity, ball skills, and balance), should be examined in the future, as we noted earlier that relationships between EF and motors skills may differ.

In this pilot study, we anticipated small effects for the rhythmic program, which were not found to be significant. As both programs did not differ on any of their characteristics other than the focus on rhythmic movement, it is possible that the dose of the rhythmic program (fourteen 30-min sessions) was not enough to create a significant difference on the motor and EF outcomes we examined. Especially given the novelty of the tasks and the fact that children perceived the rhythmic intervention as more difficult, it may be that they need a longer period of time to learn the skills and, thereby, demonstrate positive effects. This hypothesis, while clearly in need of further examination, suggests that different types of PA programs (potentially those that vary in complexity) may have a different timeline for motor and cognitive learning outcomes to be evident, which is something that should be taken into consideration when exploring the exercise–cognition relationship. Additional research is also needed regarding the potential success of the rhythmic program with children of different ages, skill levels, and cognitive development.

Our study provides evidence for the feasibility of the rhythmic program as well as recommendations for future acceptability, adaptation, and implementation of the program. Further, our study provides additional evidence related to the beneficial role of qualitatively rich PA programs that stimulate children’s motor and EF skills and supports the suitability of such programs in physical education, as highlighted by exercise and cognitive scientists (Pesce, 2012; Diamond, 2015; Schmidt et al., 2015; Pesce et al., 2019; Tomporowski and Pesce, 2019; Vazou et al., 2019b). Lastly, this study contributes to the literature by exploring the potential value of rhythmic programs as a vehicle in helping children develop motor and EF skills while deriving joy and positive social interactions from the program. Additional research is clearly warranted to better understand the effects of different types of PA programs, and particularly rhythmic programs, on motor, cold EF, and hot EF skills and social emotional factors.

## Data Availability Statement

The raw data supporting the conclusions of this article will be made available by the authors, without undue reservation.

## Ethics Statement

The studies involving human participants were reviewed and approved by the Iowa State University. Written informed consent to participate in this study was provided by the participants’ legal guardian/next of kin.

## Author Contributions

SV and AS designed the study, led the program implementation, interpreted the findings, and drafted the manuscript. BK contributed in the methodology, data collection, implementation of the programs, data processing, and helped drafting the manuscript. SV conducted the analysis. KL contributed in the methodology, interpreted the findings, and helped draft the manuscript.

## Conflict of Interest

The authors declare that the research was conducted in the absence of any commercial or financial relationships that could be construed as a potential conflict of interest.

## Footnotes

1 www.drums-alive.com
